# Evaluation of Platelet and Leukocyte Counts in Canine Platelet-Rich Plasma Obtained After Successive Blood Collections From the Same Patient and the Effects of Freezing on the Concentration of Growth Factors Present in It

**DOI:** 10.3389/fvets.2022.838481

**Published:** 2022-04-05

**Authors:** Victoria DeMello, Grace Chen, Joseph Wakshlag, David Mason

**Affiliations:** ^1^LasVegas Veterinary Specialty Center, Las Vegas, NV, United States; ^2^Department of Clinical Sciences, Cornell University College of Veterinary Medicine, Ithaca, NY, United States

**Keywords:** platelet rich plasma, freezing, growth factor concentrations, platelet concentration, dog

## Abstract

**Objective:**

The purpose of this study was 2-fold: to evaluate whether the timing of collection influences the platelet counts and leukocyte counts of PRP samples, and to evaluate growth factor concentrations in canine PRP after freezing and storage without a preservation agent for 6 months of time.

**Materials and Methods:**

Whole blood collection was performed three times over the course of 4 weeks. All PRP samples were evaluated with a CBC analysis. The PRP samples were frozen and stored without a preservation agent for the duration of the 4-week study.

**Results:**

The platelet and leukocyte counts were not statistically significant between the timing of blood draws over the course of 4 weeks. All three growth factors were present in measurable quantities after freezing and storage for 6 months without a preservation agent.

**Clinical Relevance:**

PDGF, TGF-β1, and VEGF were all present in measurable quantities. Furthermore, PDGF and TGF-β1 were correlated with platelet count of the final PRP. VEGF concentrations were able to be quantified. We correctly hypothesized growth factor concentrations would be present and measurable in canine PRP frozen and stored without a preservation agent for 6 months.

## Introduction

Platelet rich plasma (PRP) and other regenerative therapeutic resources are being studied and used in clinical situations in veterinary medicine with increasing frequency (1–6). Studies have been performed that evaluated cellular composition of PRP along with its clinical efficacy (7–11).

Platelets contain alpha granules which release growth and differentiation factors, chemokines, and cytokines. The growth factors released from activated platelets play a major role in hemostasis and wound healing. These growth factors include but are not limited to transforming growth factor (TGF-β1), vascular endothelial growth factor (VEGF), and platelet-derived growth factor (PDGF). At sites of injury, PRP entraps MSCs and supports the proliferation and differentiation of surrounding endothelial, and other stromal cells resulting in accelerated wound healing (4, 12–14).

Due to these growth factors, platelet-rich plasma (PRP) has been used to treat many medical conditions along with various orthopedic conditions including osteoarthritis (OA), tendon injuries, ligament injuries, and bone injury/remodeling in human and veterinary medicine (1).

PRP is composed of varying levels of platelets, red blood cells, and leukocytes.

The ideal composition of therapeutic canine PRP is unknown. Leukocyte-rich PRP has been found to have an increased concentration of IL-1-beta, IL-6, and TNF-alpha which are pro-inflammatory mediators produced by neutrophils (7, 8, 15). Leukocyte reduced PRP has been found to reduce inflammation and promote healing (6, 16). Although an overall increase in leukocyte concentration in PRP is suspected to be deleterious, an increase in lymphocyte concentration is suspected to have healing properties when present in PRP (17); however further studies evaluating lymphocyte rich PRP are needed.

Several studies have been performed to evaluate the processing, final composition, and efficacy of PRP products. These studies compared commercially available PRP preparation kits to determine which kit had the highest platelet concentration while reducing the concentration of red blood cells in the final PRP product (10, 16).

Stief et al. quantified numerous growth factors in autologous canine plasma (ACP), but with ELISA tests not validated for canine patients (18). They showed an average TGF -β1 concentration of 2,000 pg/ml. This was limited by an upper limit of quantification of the aforementioned ELISA assay. Franklin et al. evaluated TGF -β1 in canine ACP using validated assays and showed a significant effect of intentional activation on TGF -β1 concentration with either CaCl_2_ or thrombin (9). These levels were correlated with platelet concentration. To the authors' knowledge, there does not appear to be any information in canines assessing the repeatability of PRP samples from patients over time and likewise there is limited information on growth factor content.

PRP processing can be expensive and time consuming leading to a need for appropriate methods of preparation and storage. Human PRP has been shown to have stable growth factor concentrations at room temperature for a short duration (<4 h) (19). In equine medicine, it has become commonplace to freeze and store PRP for later use. A prospective evaluation of freezing conditions and duration of storage on growth factors in equine PRP revealed stable concentration(s) of growth factors (PDGF and TGF-β1) after freezing for 6 months in liquid nitrogen (20). Another study evaluating growth factor concentrations in human PRP after freezing and storage without a preservation agent, revealed an overall decrease in growth factors assessed (21, 22). In clinical practice, the ability to store samples in temperatures below −20°C is usually not feasible and preservation of growth factors under normal freezing conditions is unknown.

The purpose of this study was 2-fold: to evaluate whether the timing of collection influences the platelet counts and leukocyte counts of PRP samples and evaluation of growth factor concentrations in canine PRP over time after freezing and storage without a preservation agent for 6 months of time.

We hypothesized that the platelet count would not change with time of collection and preparation. Furthermore, the growth factor concentrations of the PRP samples would be measurable and consistent with prior literature after freezing at −20°C and subsequent thawing of the samples.

## Materials and Methods

This prospective study was performed from October 2019 to January 2020 at Las Vegas Veterinary Specialty Center (LVVSC). Fifteen healthy adult canine patients were recruited for this study. The patients' age ranged from 15 months to 12 years of age with an average of 5 years of age. The weight of the patients ranged from 17 to 70 Kg with an average weight of 36.9 Kg. All animals were evaluated to be free of medical problems as determined by physical exam (performed by a veterinary surgical intern), clinical history, and complete blood count (CBC) analysis. Eight of the study participants were spayed females and seven were neutered males. None of the animals participating in the study received any prescription medications during the study period. Results of the CBC analysis were required to be within respective reference ranges for each cell type.

In accordance with the Association for Assessment and Accreditation of Laboratory Animal Care (AAALAC) International Rules of Accreditation, this study was performed with the approval of the LVVSC Research Committee and with signed owner consent. All dogs were directly overseen by a veterinarian to ensure no harm was incurred during study participation, and the owners were present at all times.

Whole blood collection was performed three times over the course of 4 weeks: Initial blood draw (time 0), 2 weeks post-initial blood draw (time 1), 4 weeks post-initial blood draw (time 2).

CBC analysis was performed on all whole blood and PRP samples with a benchtop hematology analyzer (Heska HT5). This benchtop analyzer uses impedance technology for counting platelets which has been determined to be the most reliable automated analysis of canine platelets (10).

The blood collection for PRP preparation was performed following the manufacturer's instructions^1^.

The system utilized in this study was the Companion Regenerative Therapies Pure-PRP II kit. This consists of a double centrifugation method using 50 ml whole blood which produces a final product of 4 ml of pure-PRP.

Fifty milliliters of whole blood was aseptically collected from the jugular or cephalic vein of the patients. The whole blood was aspirated into a 60 ml syringe while rocking the syringe carefully to allow for mixing of the blood with 10 ml Anticoagulant Citrate Dextrose, Solution A (ACD-A). None of the patients were sedated for the phlebotomy procedure. Whole blood was immediately evaluated with a CBC analysis (15 μl used per CBC analysis) and processed for PRP. The PRP method includes a double centrifugation method totaling 6 min and a total preparation time of 10 min (not including blood sample collection). Post-preparation, all PRP samples were evaluated with a CBC analysis and then stored. The PRP samples were frozen and stored without a preservation agent using a −20°C freezer for the duration of the 4-week study. Baseline growth factor concentrations were not assessed in the samples prior to freezing. The samples were then transferred on dry ice to the laboratory of analysis and stored for the remainder of the 6 months at −20°C in a non-automated defrosting freezer prior to growth factor concentration assessment. All samples were thawed on ice and subjected to centrifugation at 10,000 g for 10 min. Supernatants were then utilized for ELISA testing for VEGF, TGF-β1, and PDGF-BB (R and D Biosystems, Minneapolis, MN). The VEGF ELISA upper and lower limits of detection were 19.5–2,500 pg/ml with an intraassay and interassay coefficient of variation of 5 and 7.5%, respectively. The TGF-B1 ELISA upper and lower limits of detection were 31–2,000 pg/ml with an intra-assay and inter-assay coefficient of variation of 2.8 and 8%, respectively. The PDGF-BB was a human kit, and a standard curve was facilitated utilizing a canine recombinant PDGF-BB (Genorise, Glens Mills, PA) using techniques from Birdwhistle et al. The upper and lower limits of detection for the PDGF ELISA were 31.2–2,000 pg/ml. The intra-assay and inter-assay coefficient of variation for PDGF-BB were 4.0 and 5.7%, respectively. All ELISAs have been validated for use in PRP samples and methods implored were based on prior publication (23).

## Statistical Analysis

Data was assessed for normality using a Shapiro-Wilks test for all cellular counts in the PRP across time and the data was found to by normally distributed for lymphocytes and platelets therefore a one-way analysis of variance was used to assess differences over time. Red blood cell and neutrophil counts displayed skewed data across time points and failed normality testing therefore a Friedman's test was utilized to assess differences across time. Normality testing using a Shapiro-Wilk test for the growth factor data over time showed that PDGF and TGF-B data was normally distributed hence a one-way ANOVA was used to assess the data, while VEGF was not normally distributed therefore a Friedman's test was implored. All statistical significance was set at a *p-*value of *P* <0.05 for all statistical testing.

To identify the correlations between platelet recovery and growth factor concentrations all 45 samples across time points were assessed using a Pearson correlation test. Correlation significance was set at a *p-*value of < 0.05. Correlation strength was based on R values obtained from the testing with an *R* < 0.3 as a poor correlation, *R* > 0.3 to 0.5 as a weak correlation, *R* > 0.5–0.7 as a moderate correlation and *R* > 0.7 as a strong correlation.

## Results

The platelet counts of the final PRP product was not statistically significant between the timing of blood draws and PRP preparation over the course of 4 weeks (Table 1). The leukocyte count overall (lymphocytes, neutrophils) and red blood cell counts within the final PRP product were not statistically significant over the three time points (Table 1). Growth factor concentrations (PDGF, VEGF, and TGF-β1) showed no differences across time points (Table 1). In the VEGF assay three of the dogs showed concentrations below the lower limit of detection and were therefore dropped from the analysis with only 12 dogs represented in the VEGF data set.

**Table 1:** Average cell counts and growth factor concentrations and standard deviation.

**Cell/Growth factor average (+SD)**	**Day 0**	**Day 14**	**Day 28**	***P*-value**
Neutrophils (10∧3/ul)	2.24 (2.54)	2.27 (3.80)	4.37 (4.53)	0.94
Lymphocytes (10∧3/ul)	7.88 (4.96)	8.22 (4.95)	9.60 (7.13)	0.54
Platelets (10∧3/ul)	1923.60 (504.35)	1911.33 (542.80)	1859.07 (487.13)	0.83
Red blood Cells (10∧6/ul)	0.22 (0.22)	0.29 (0.33)	0.72 (0.96)	0.21
VEGF (pg/ml)	36.46 (21.35)	37.58 (26.80)	64.17 (75.29)	0.16
PDGF (pg/ml)	71253.80 (2063.93)	81150.60 (1668.10)	82097.13 (1382.24)	0.09
TGF-β (pg/ml)	67995.00 (21908.57)	59772.80 (28732.75)	59229.10 (35338.75)	0.81

*The average cell count (neutrophils, lymphocytes, platelets, red blood cells) and growth factor concentrations (PDGF, TGF-β1) in PRP from 15 dogs over 4 weeks. Data was assessed for normality using a Shapiro-Wilks test for all cellular counts. Red blood cell and neutrophil counts failed normality testing; therefore, a Friedman's test was used. Normality testing using a Shapiro-Wilk test for the growth factor data showed PDGF and TGF-B data was normally distributed hence a one-way ANOVA was used to assess the data. VEGF was not normally distributed therefore a Friedman's test was used. All statistical significance was set at a p-value of P < 0.05 for all statistical testing*.

**Figure 1:**
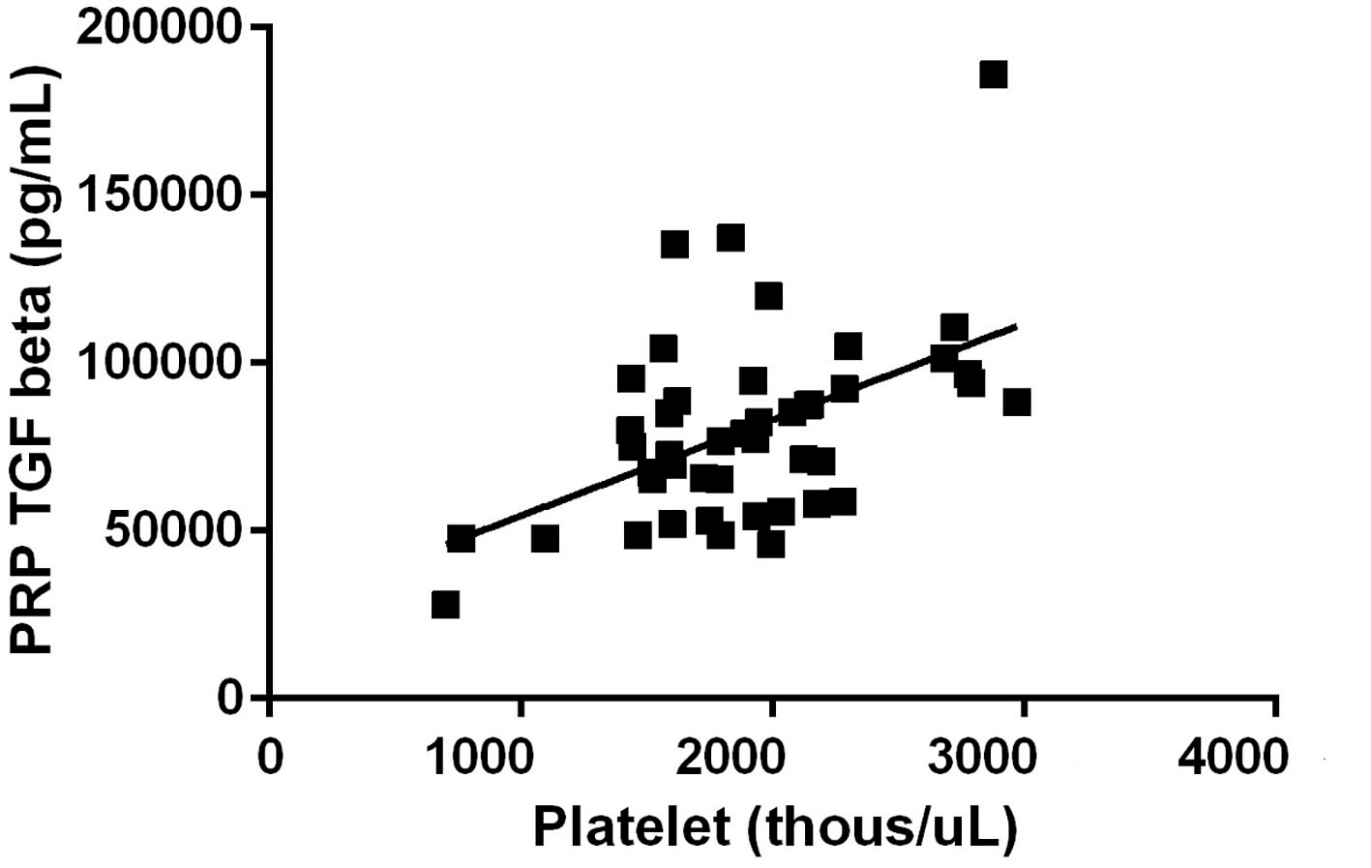
Transforming Growth Factor- Beta concentration correlation to the concentration of platelets. TGF-β1 concentration was observed to have a positive correlation between platelet concentration growth factor concentration in the final PRP product (*R* = 0.51, *p* < 0.01).

**Figure 2:**
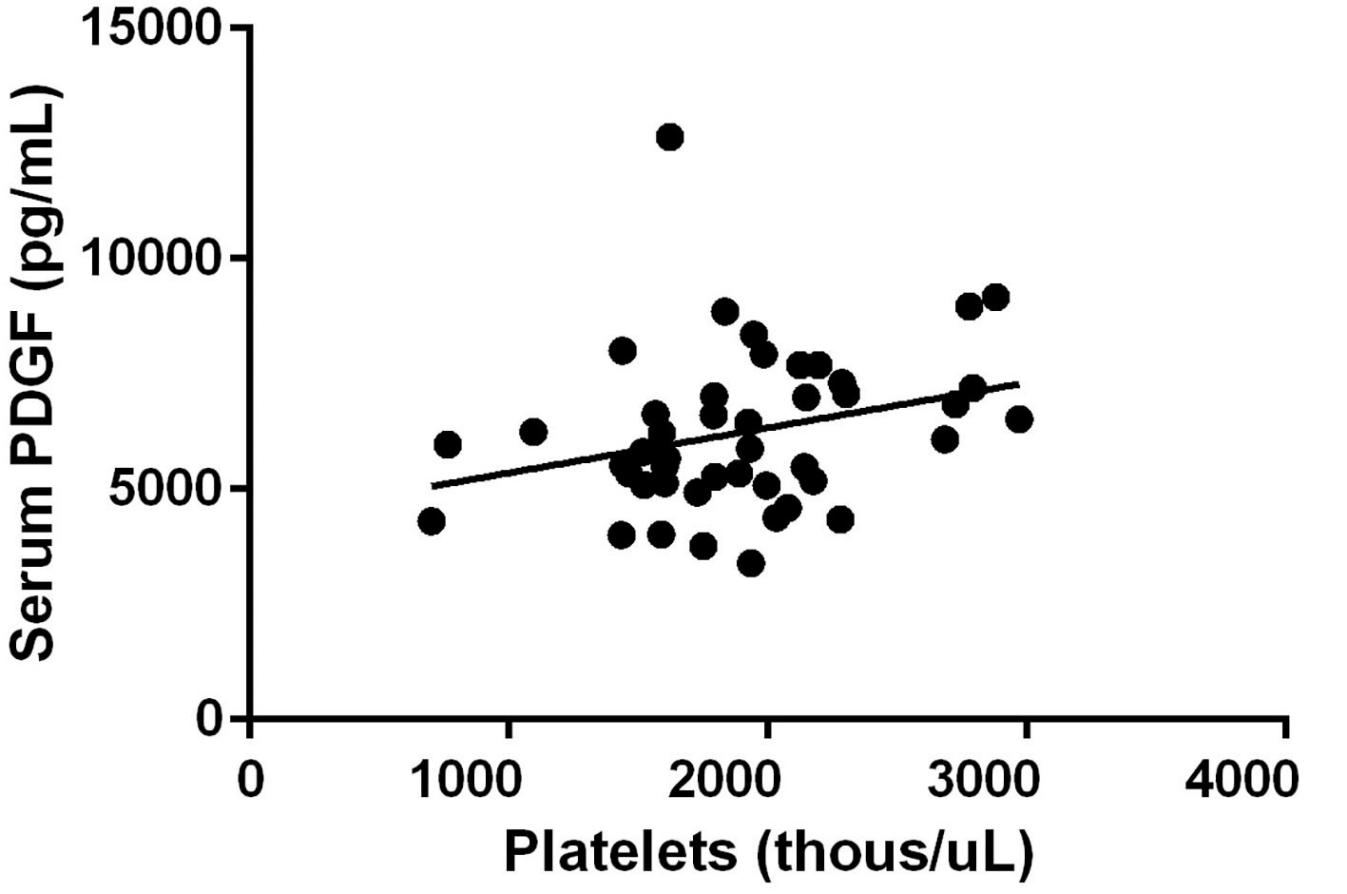
Platelet Derived Growth Factor concentration correlation to the concentration of platelets. PDGF concentration was also observed to have a positive correlation between platelet concentration growth factor concentration in the final PRP product (*R* = 0.29, *p* = 0.06).

All three growth factors were present in measurable quantities. TGF-β1 concentration was observed to have a positive correlation between platelet concentration growth factor concentration in the final PRP product (*R* = 0.51, *p* < 0.01) (Figure 1). PDGF concentration was also observed to have a positive correlation between platelet concentration growth factor concentration in the final PRP product (*R* = 0.29, *p* = 0.06) (Figure 2). VEGF concentrations were detectable in the final PRP product but were universally low compared to TGF-β1 and PDGF. VEGF was not observed to be correlated with platelet levels within the final PRP sample (*R* = 0.036, *p* = 0.51).

## Discussion

To the authors' knowledge, no information evaluating the consistency of platelet and leukocyte counts within PRP samples over a period of time is available. Our study used a single commercially available PRP kit that has been determined to create a leukoreductive and high yield platelet concentration PRP product (16). We evaluated platelet count and individual leukocyte counts in the anticoagulated blood and subsequent PRP samples using an automated CBC analysis. The platelet and leukocyte counts of the final PRP product were compared across three time points (initial blood draw, 2 weeks post-initial blood draw, 4 weeks post-initial blood draw). No statistical difference was observed within these counts over the course of the study suggesting that PRP preparation is unlikely to be influenced by time or the kits utility on a daily basis showing consistency in product delivered. Of course, overall platelet counts are very important and before implementing PRP, a platelet count may aid in decision making regarding efficacy of PRP in each individual patient. Furthermore, our study confirmed previously published results using this kit and its overall consistency in producing an overall leukoreductive (lymphocyte rich) high platelet yield product (16).

An additional goal of our study was to quantify growth factor concentrations after freezing and long-term storage (6 months) without a preservation agent. In a study performed by Franklin et al., five different kits were directly compared evaluating their ability to concentrate growth factors. Canine PRP was prepared using the manufacturer's instructions in which case, one system out of five included instructions for use of CaCl_2_ for platelet activation (9). This study found concentrations of TGF-β1 in the final PRP product of all systems. PDGF concentrations were only identified in PRP samples that underwent platelet activation. None of the systems assessed displayed measurable quantities of VEGF (lower limit of assay 39.1 pg/ml). This study concluded a weak positive correlation between platelets and TGF if the platelets were not activated.

The results of our study differ from the Franklin et al. study as PDGF, TGF-β1, and VEGF were all present in measurable quantities. Furthermore, TGF-β1 and PDGF were correlated with platelet concentration of the final PRP product although only weak to moderately. Interestingly, VEGF concentrations were able to be quantified in 12 of the 15 dogs utilizing this particular assay; however, at very low levels. The low concentration of VEGF observed in our study and the non-measurable concentration in studies previously (9) performed could indicate canine platelets may inherently have low concentrations of VEGF or none at all, making canine platelets different from other species. Further analysis evaluating VEGF through other techniques including proteomic analysis of western blotting techniques to evaluate canine platelets are warranted.

Several studies have been performed evaluating the effect of freezing and subsequent thawing on equine and human PRP (19, 20, 24). McClain and McCarrell evaluated the effects of freezing on equine PRP growth factor concentrations growth factors (TGF and PDGF) without a preservation agent. The PRP samples in this study were all activated with CaCl_2_ and bovine thrombin prior to growth factor quantification after thawing (20). The process of freezing can cause an increase in growth factor concentration through cold-induced platelet activation as well as potential rupture of platelets and alpha-granules (25). For these reasons and the practicality of using PRP in general clinical practice, we utilized only freeze thawing techniques to examine the integrity of the growth factors after 6 months of storage. The clinical significance of the effects of freezing on canine PRP is unknown. Initial evaluation of growth factors was not performed in our study which would have strengthened our findings regarding degradation of these growth factors under normal clinical freezing conditions. However, our finding of ample PDGF and TGF-β1 in our samples comparable to concentrations observed under other activating conditions by Franklin et al. suggests that these growth factors appear to survive long term storage at −20°C which is encouraging from a clinical perspective regarding storage for future use.

Further limitation of our study includes the small sample size in the number of canine PRP samples assessed. A study including a larger canine population to evaluate growth factor concentrations in frozen-thawed samples is indicated with comparisons to baseline growth factor concentrations. Additionally, the value of platelet activation in evaluating growth factor concentration could not be assessed as platelet activation was not indicated within manufacturer's instructions for PRP preparation. It is possible that platelet activation during the PRP preparation or post-thawing may further increase growth factor concentrations and the importance for intact platelet or activated platelets during injection has not been studied clinically. Furthermore, the therapeutic level of growth factor concentrations, platelet concentrations, or leukocyte concentrations is currently unstudied regarding efficacy and longevity of clinical response regarding PRP.

The goal of this study was to evaluate changes in platelet counts in PRP over time in a dog population. We correctly hypothesized that no correlation involving platelet count and date of PRP preparation would be observed. Additionally, we hypothesized growth factor concentrations would be present and measurable in canine PRP frozen and stored without a preservation agent for 6 months.

Further evaluation determining the therapeutic level of growth factor concentrations in PRP and determining if activation of platelets post-long-term storage will further increase growth factor concentrations is needed.

## Data Availability Statement

The raw data supporting the conclusions of this article will be made available by the authors, without undue reservation.

## Ethics Statement

The animal study was reviewed and approved by LVVSC Research Committee. Written informed consent was obtained from the owners for the participation of their animals in this study.

## Author Contributions

DM contributed to experimental design, funding, and manuscript preparation. VD performed data collection and manuscript preparation. GC performed data collection. JW performed data evaluation and assisted in manuscript preparation. All authors contributed to the article and approved the submitted version.

## Conflict of Interest

The authors declare that the research was conducted in the absence of any commercial or financial relationships that could be construed as a potential conflict of interest.

## Publisher's Note

All claims expressed in this article are solely those of the authors and do not necessarily represent those of their affiliated organizations, or those of the publisher, the editors and the reviewers. Any product that may be evaluated in this article, or claim that may be made by its manufacturer, is not guaranteed or endorsed by the publisher.

## Footnotes

^1^CRT PurePRP, Canine Regenerative Therapies, Fort Meyers, FL, USA.
